# Genome-wide characterization of the accessible chromatin regions in chickpea

**DOI:** 10.1186/s12864-026-13136-5

**Published:** 2026-07-03

**Authors:** Qi Liang, Lang Yang, Ling Zhang, Xingbin Lv, Shuyun Tian, Ying Xiong, Zhiyi Zhang, Mingkun Huang, Hua Yang, Yufang Hu

**Affiliations:** 1 School of Life Sciences, Nanchang University, 999 Xuefu Avenue, Honggutan New District, Nanchang, 330047 China; 2 Jiangxi Provincial Key Laboratory of Ex Situ Plant Conservation and Utilization, Lushan Botanical Garden, Chinese Academy of Sciences, 9 Zhiqing Road, Jiujiang, 332900 China; 3 School of Pharmacy, Jiangxi University of Traditional Chinese Medicine, 1688 Meiling Avenue, Xinjian District, Nanchang, 330004 China

**Keywords:** *Cicer arietinum*, Accessible chromatin region, ATAC-seq, Histone modification, Epigenetics

## Abstract

Chromatin accessibility is important for genome architecture and gene expression in plants. In this study, the ATAC-seq method was utilized to identify accessible chromatin regions (ACRs) across the chickpea (*Cicer arietinum*) genome, an important legume crop cultivated worldwide. A total of 11,555 ACRs were identified in the chickpea genome, enriched at gene transcription start sites (TSS) and positively correlated with gene expression. Furthermore, as expected, a number of known transcription factor (TF) binding motifs were enriched in these ACRs. Integration of histone modification data, ACRs were found to be closely associated with H3K27ac and H3K4me3 modifications to regulate gene expression. In addition, the gain and loss of ACRs has been demonstrated to have significant effects on the expression of homologous genes. Collectively, this study provides a comprehensive understanding of the genomic function of ACRs in chickpea.

**Supplementary Information:**

The online version contains supplementary material available at https://doi.org/10.1186/s12864-026-13136-5.

## Introduction

Chromatin, a complex structure composed of DNA and proteins, is formed by the binding of DNA to histones to form nucleosomes, which are further folded and compacted [1–3]. These structures undergo dynamic changes during DNA replication or transcription, allowing the tight structure of chromosomes to become open [4, 5]. The accessible chromatin regions (ACRs) in the genome facilitate the binding of trans-acting factors to the *cis*-regulatory elements present in promoters, enhancers, insulators, and silencers. This property, which allows for the binding of transcription factors (TFs), is defined as chromatin accessibility [6–8]. In addition to being regulated by TFs, the chromatin is also subject to various histone modifications, which affect the chromatin structure and the subsequent gene expression [9, 10]. Currently, ATAC-seq (Assay for Transposase-Accessible Chromatin with high throughput sequencing) [4, 11], as a common method for ACR analysis, uses the transposase Tn5 [12], an enzyme capable of labeling ACRs, to fragment these sequences for library construction to improve the understanding of the regulatory mechanisms of gene expression across plant genomes.

*Cicer arietinum* (chickpea) is an herbaceous plant, belonging to the genus *Cicer* in the family Fabaceae. It is an important food crop in China, is rich in protein, various amino acids, vitamins, crude fiber, calcium, magnesium, iron, and other ingredients, and has a high economic value and extensive application prospects [13, 14]. However, studies on regulatory sequences in chickpea have remained limited. In this study, we have adopted ATAC-seq to identify the ACRs across the chickpea genome. When integrated with other data, our results provided a comprehensive understanding of the genomic features of these important *cis*-regulatory sequences and their impact on downstream gene expression. Furthermore, our data provided genomic resources for further exploration of non-coding regulatory ACRs for genetic diversity and crop improvement in chickpea.

## Material and method

### Plant materials

Chickpeas (*Cicer arietinum cv. PBA*) were grown naturally in a greenhouse at 26 °C and 65% relative humidity, and leaf samples were collected 15 days after germination for subsequent experiments.

### RNA-sequencing (RNA-seq) data analysis

Approximately 0.1 g of chickpea leaf sample (collected as described above) was flash-frozen in liquid nitrogen and ground to powder. Total RNA was extracted using the RNeasy Plant Mini Kit (Qiagen) following the manufacturer’s protocol. Extracted total RNA was treated with RNase-free DNase I (Thermo Fisher Scientific) to remove genomic DNA and subsequently purified. RNA purity and integrity were assessed using a NanoDrop 2000 spectrophotometer and an Agilent 2100 Bioanalyzer, respectively. Only samples with an RNA Integrity Number (RIN) value ≧ 7.0 were used for library construction. For library construction, the VAHTS Universal V10 RNA-seq Library Prep Kit for Illumina was used following the manufacturer’s protocol. Adapter trimming of the raw data was performed using TrimGalore (http://github.com/FelixKrueger/TrimGalore, default parameters), and the resulting reads were then mapped to the chickpea reference genome [15] using the HISAT-StringTie pipeline [16]. Gene expression levels were calculated using the default parameters and expressed as fragments per kilobase per million mapped reads (FPKM).

### Assay for transposase-accessible chromatin using sequencing (ATAC-seq)

Leaf ATAC-seq library construction was performed as previously reported [17, 18]. Briefly, approximately 0.3 g of chickpea leaf sample was flash-frozen in liquid nitrogen, and then rapidly ground to powder. Nuclei were extracted by adding 10 mL of lysis buffer, followed by a washing step with 1 mL of Wash Buffer I and Wash Buffer II, respectively. Subsequently, about 10^5^ nuclei were resuspended in 50 µL of Tn5 reaction solution (TruePrep DNA Library Prep Kit V2 for Illumina, Vazyme, TD501) and incubated at 37 °C for 30 min. The tagged DNA was purified using the DNA Clean & Concentrator Kit (Zymo Research, D4014) and amplified using Q5 DNA polymerase (NEB, M0491) with indexed primers from the TruePrep Index Kit V2 for Illumina (Vazyme, TD202). The library was sent for sequencing on the Illumina NovaSeq PE150 platform.

Raw ATAC-seq data were filtered by TrimGalore and mapped to the chickpea genome by Bowtie2 [19] with the parameter set as: -X 1000. Samtools [20] was used to extract the reads with the MAPQ value above 30, which were further used for peak calling by MACS3 (parameters set as “--nomodel --shift 100 --extsize 200”) [21]. Chickpea genomic DNA, processed by the Tn5 enzyme, was used as an input control. The reproducible peaks between two replicates were defined as ACRs. Annotation of the ACRs was performed using the annotatePeaks.pl command in HOMER (http://homer.ucsd.edu/homer) with the CDC Frontier v2.0 genome and GFF annotation. Visualization of ATAC-seq signals was performed using Deeptools [22] and IGV [23]. MEME Suite [24] was used for motif analysis.

### Histone chromatin immunoprecipitation sequencing (ChIP-seq)

Chickpea histone ChIP-seq was performed as described in the previous study with minor modifications [25–27], using ChIP-seq grade anti-H3K27ac (PTMA, PTM-116) and anti-H3K4me3 (PTMA, PTM-613) antibodies. Approximately 5 ng of ChIP-seq DNA and input DNA were utilized for library construction using the TruePrep DNA Library Prep Kit V2 for Illumina (Vazyme, TD501). Adapter trimming and removal of low-quality reads were performed using Trim_Galore, and mapping of reads to the genome was performed using Bowtie2 [19]. The input library was used as a control. Mapped reads (MAPQ > 30) were peak-called using MACS3 with the narrow peak model [28].

## Results

### Identification of ACRs in the chickpea genome by ATAC-seq

**Fig. 1:**
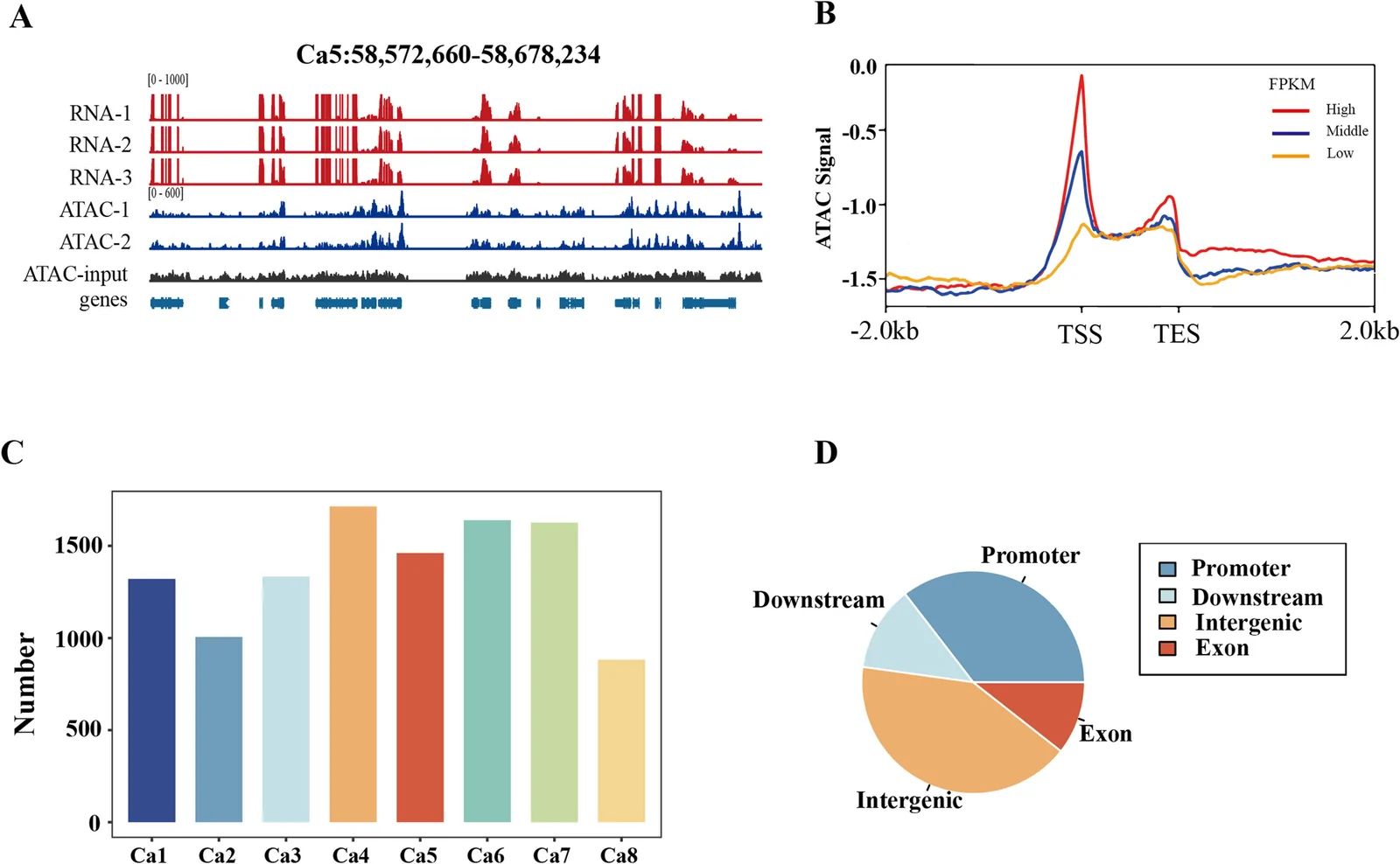
Data summary of ATAC-seq in chickpea genome. **A** Overview of RNA-seq and ATAC-seq signal coverage, using the region 58,572,660 − 58,678,234 in chromosome 5 (Ca5) as an example. **B** Enrichment of ATAC-seq signal and a positive correlation with the gene expression levels are observed. The profile plot displays signal in the ± 2 kb regions flanking the TSS/TES, with the gene body scaled to 1 kb. TSS, transcription start site; TES, transcription end site. High, Middle, and Low indicate the high (FPKM ≥ 10, *n* = 10463), medium (1 ≤ FPKM < 10, *n* = 6727), and low (FPKM < 1, *n* = 7915 ) expression genes, respectively. **C** The number of ACRs in each chromosome. Ca1-Ca8 indicates the 8 chromosomes observed in chickpea. **D** Distribution of ACRs in the chickpea genome, including Promoter (35%), Downstream (12%), Intergenic (42%), and Exon (11%)

In this study, ATAC-seq was employed to identify and characterize ACRs across the chickpea genome. Leaf ATAC-seq libraries from two biological replicates of chickpea generated approximately 99.6 million (M) and 100.8 M reads, respectively (Figure S1, S2, and S3, Table S1). As expected, ATAC-seq signals were mainly enriched around the transcription start site (TSS) and exhibited a positive correlation with the expression levels of coding genes (Fig. 1A and B), indicating that the chromatin accessibility is associated with gene activation, which is consistent with previous reports. A total of 11,555 ATAC-seq enriched regions were identified in two replicates and defined as ACRs, which were found to be associated with 7,609 coding genes (Fig. 1C, Table S2). Genomic annotation of these ACRs revealed that the majority of ACRs were in the promoter (35%) and the distal intergenic regions (42%), while 12% of ACRs were observed in the downstream region and 11% in the exonic region (Fig. 1D, Table S2).

### Enrichment analysis of conserved transcription factor motifs enrichment in the ACRs

**Fig. 2:**
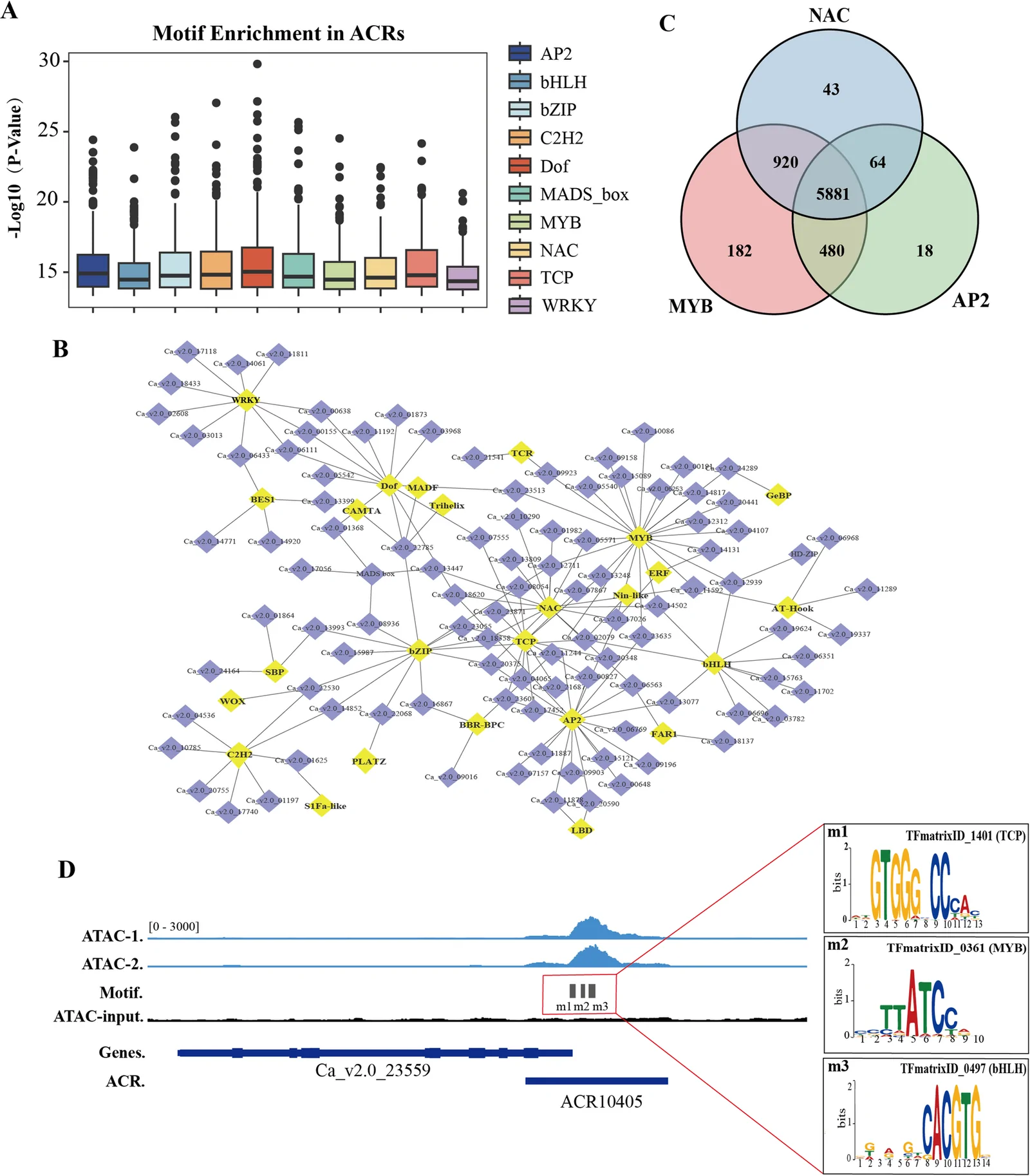
Conserved transcription factor motifs enriched at ACRs in chickpea genome. **A** Top ten enriched transcription factor (TF) motifs detected in ACRs, including AP2, bHLH, bZIP, C2H2, Dof, MADS_box, MYB, NAC, TCP, and WRKY motifs. **B** Potential TF regulatory network predicted from genes associated with ACR enriched motifs. Network construction logic: TFs (identified by FIMO scan in ACRs) were linked to the nearest gene associated with that ACR, and the network was visualized in Cytoscape.Yellow squares indicate corresponding TFs and purple squares indicate associated genes. **C** Number of potential co-targeted genes by the top three enriched TF motifs (MYB, NAC, and AP2) in ACRs. **D** Examples of TF motifs at an ACR, such as TCP (TFmatrix_ID1401), MYB (TFmatrix_ID0361), and bHLH (TFmatrix_ID0497)

TFs can regulate gene expression by recognizing specific DNA sequences. During chromatin opening, TFs bind to DNA sequences (motifs) in the open chromatin regions. Therefore, ACRs tend to be enriched in conserved TF binding motifs [29, 30]. To assess the enrichment of TF binding motifs on chickpea ACRs, the TF binding motifs reported in the PlantPAN3.0 database were mapped to these ACRs [31]. The result showed that conserved TF binding motifs were significantly enriched on ACRs, with the top 10 enriched motifs being AP2, bHLH, bZIP, C2H2, Dof, MADS_box, MYB, NAC, TCP and WRKY (Fig. 2A and D, Table S3). Furthermore, aggregated Tn5 insertion profiles provided evidence of TF occupancy at these enriched motifs (Figure S4). Subsequently, a potential TF regulatory network was constructed using TF binding motifs enriched in ACRs and genes related to the downstream of ACRs (Fig. 2B). In addition, a total of 5,881 (77.5%) genes were observed to be co-targeted by the MYB, NAC, and AP2 families (Fig. 2C). Taken together, these results suggest that there are several conserved TF-binding motifs in chickpea ACRs that could synergistically regulate the expression of downstream genes.

### Histone modifications in ACRs

**Fig. 3:**
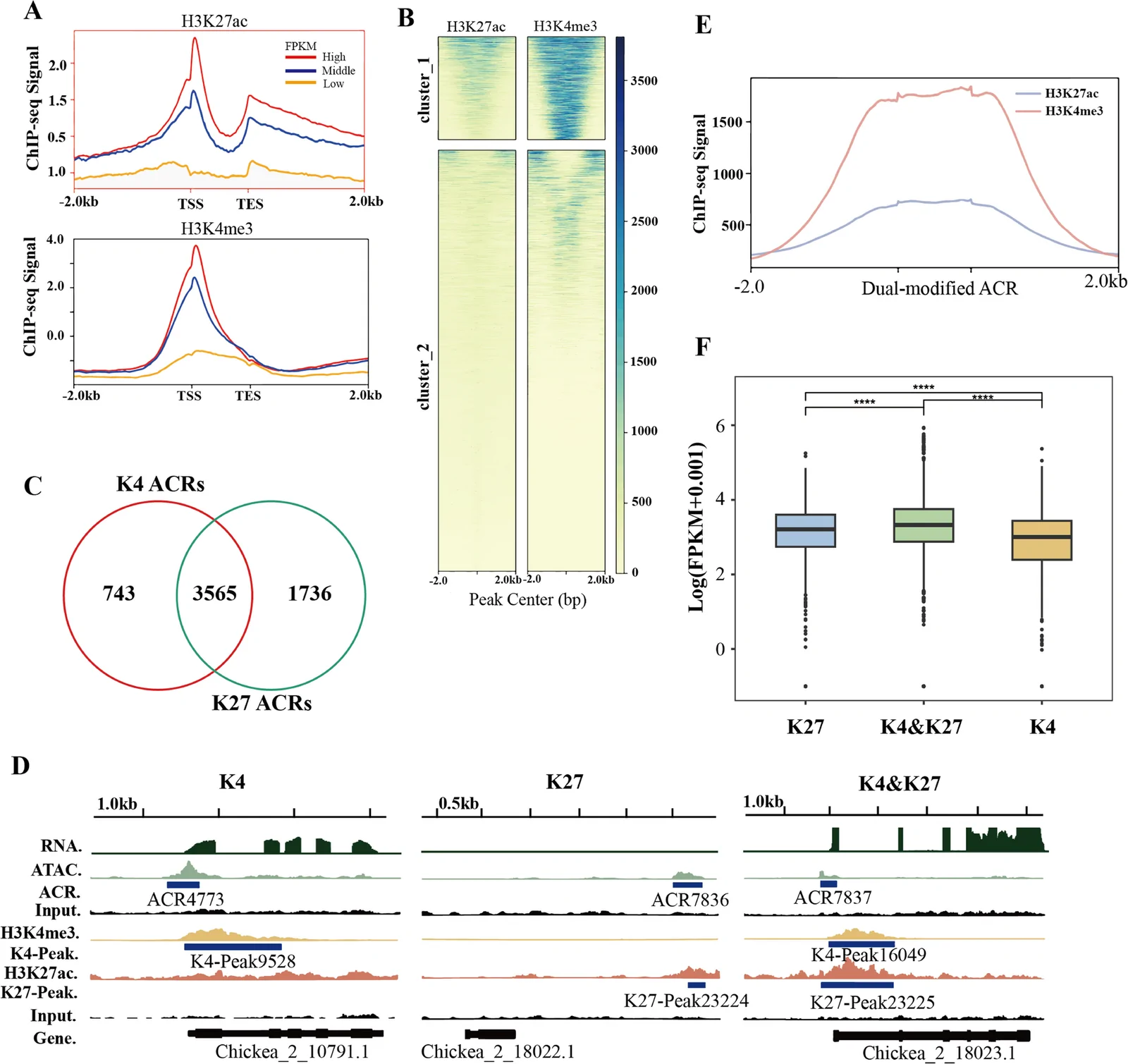
Histone modifications features at ACRs in chickpea genome. **A** Histone ChIP signal enrichments of H3K27ac and H3K4me3. The profile plot displays signal in the ± 2 kb regions flanking the TSS/TES, with the gene body scaled to 1 kb. High, Middle, and Low indicate the high (FPKM ≥ 10, *n* = 10463), medium (1 ≤ FPKM < 10, *n* = 6727), and low expression (FPKM < 1, *n* = 7915). TSS, transcription start site; TES, transcription end site. **B** Heatmap showing the ACRs are classified into two major clusters according to the H3K27ac and H3K4me3 signal intensities by the K-mean algorithm. **C** Venn diagram showing that 3,565 ACRs are dual-modified by H3K4me3 (K4) and H3K27ac (K27). **D** Examples of the H3K4me3 (K4), H3K27ac (K27) modified ACRs, K4 and K27 dual-modified ACRs. **E** H3K4me3 has more signal coverages than H3K27ac at K4 and K27 dual-modified ACRs. **F** Expression levels of genes associated with K4 and K27 dual-modified ACRs are significantly higher than those with either K27 or K4 modification only (Kruskal-Wallis test; **** *p* < 0.0001)

It has been reported that histone modifications are usually associated with ACRs to regulate gene expression [32–35]. In this study, ChIP-seq was adopted to investigate the distribution of H3K27ac and H3K4me3 in chickpea leaf ACRs and the relationship between these histone modifications and ACRs. A total of 32,260 H3K27ac-enriched peaks and 22,164 H3K4me3-enriched peaks were identified in chickpea (Tables S4 and S5). High Pearson correlation coefficients between biological replicates demonstrated the consistency of our ChIP-seq data (Figure S5). H3K4me3- and H3K27ac-modified ACRs were mainly enriched in the promoter region, and positively correlated with gene expression as expected (Fig. 3A and S6, Table S4 and S5).

Using the K-mean algorithm, the histone-modified ACRs could be classified into two major clusters (Fig. 3B). The results indicated that the majority of these ACRs were modified by H3K4me3, with a smaller number modified by H3K27ac in Cluster I, whereas most of the modified ACRs in Cluster II exhibited minimal modified signal. Furthermore, it was observed that 743 and 1,736 ACRs were modified only by H3K4me3 and H3K27ac, respectively, with 3,565 ACRs being dual-modified by these two histone marks (Fig. 3C and D, Table S6). Interestingly, in the dual-modified ACRs, the overall signal coverage of H3K4me3 was higher than that of H3K27ac (Fig. 3E). The expression of genes associated with dual-modified ACRs was higher than that of those with only a single modification (Fig. 3F, Table S6). Taken together, these data suggest that dual modifications of H3K27ac and H3K4me3 at ACRs may play an important role in chromatin opening and gene activation.

**Fig. 4:**
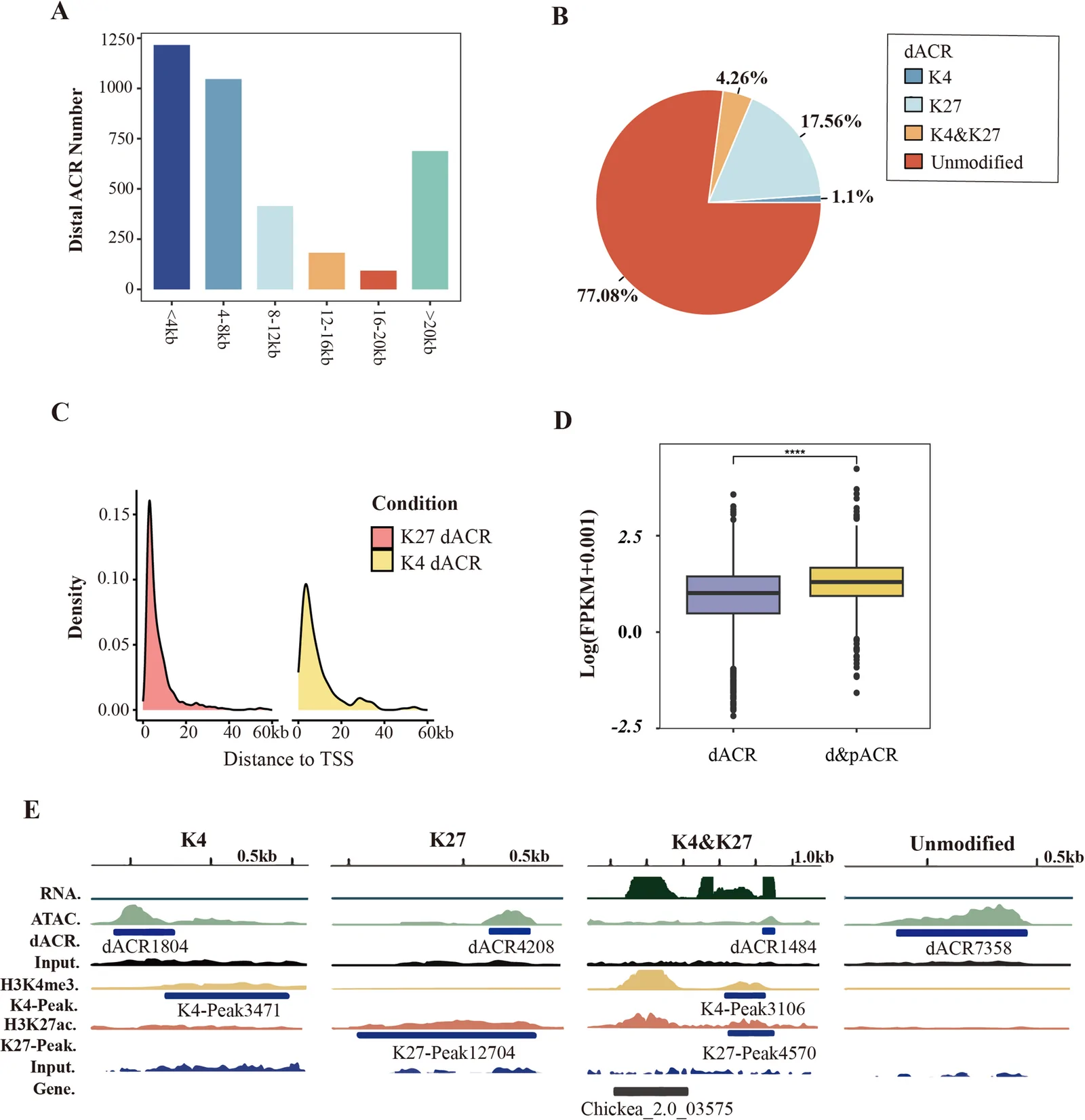
Characterization of distal ACRs (dACRs) in chickpea genome. **A** The distance from dACRs(*n* = 3639) to their nearest associated genes. **B** The percentage of unmodified (*n* = 2805), H3K4me3 (K4) modified (*n* = 40), H3K27ac (K27) modified (*n* = 639), K4 and K27 dual-modified (*n* = 155) dACRs. **C** The distribution of K4 and K27 modified dACRs according to the distance to the transcription start site (TSS). **D** The expression levels of genes associated with both pACRs and dACRs (*n* = 1759) were significantly higher than those with only dACRs (*n* = 557) (Wilcoxon test; *p* < 0.0001). **E** Examples of K4 modified, K27 modified, K4 and K27 dual-modified dACRs, as well as the unmodified dACRs

Distal ACRs (dACRs, Distance to TSS > 2000 bp), located in intergenic regions, might act as enhancers and play an important role in regulating gene expression [36, 37]. In this study, a total of 3,639 dACRs were identified and mainly distributed in the 2–8 kb intergenic region in chickpea. In addition, 688 dACRs were detected in regions more than 20 kb away from the nearest TSS (Fig. 4A, Table S2). In contrast to the proximal ACRs (pACRs, Distance to TSS≦2000 bp), the majority of the dACRs (77.08%) were found to be unmodified by either H3K4me3 or H3K27ac (Fig. 4B and E, Table S4 and S5). The dACRs with H3K4me3 or H3K27ac modification were located in the regions close to the TSS (< 10 kb) (Fig. 4C). As previously reported, the expressions of genes associated with both dACRs and pACRs were significantly higher than those associated with dACRs alone, indicating that dACRs might act as an enhancer partner, interacting with pACRs to promote gene expression (Fig. 4D).

### Effects of ACRs absence on the homologous gene expression in chickpea

**Fig. 5:**
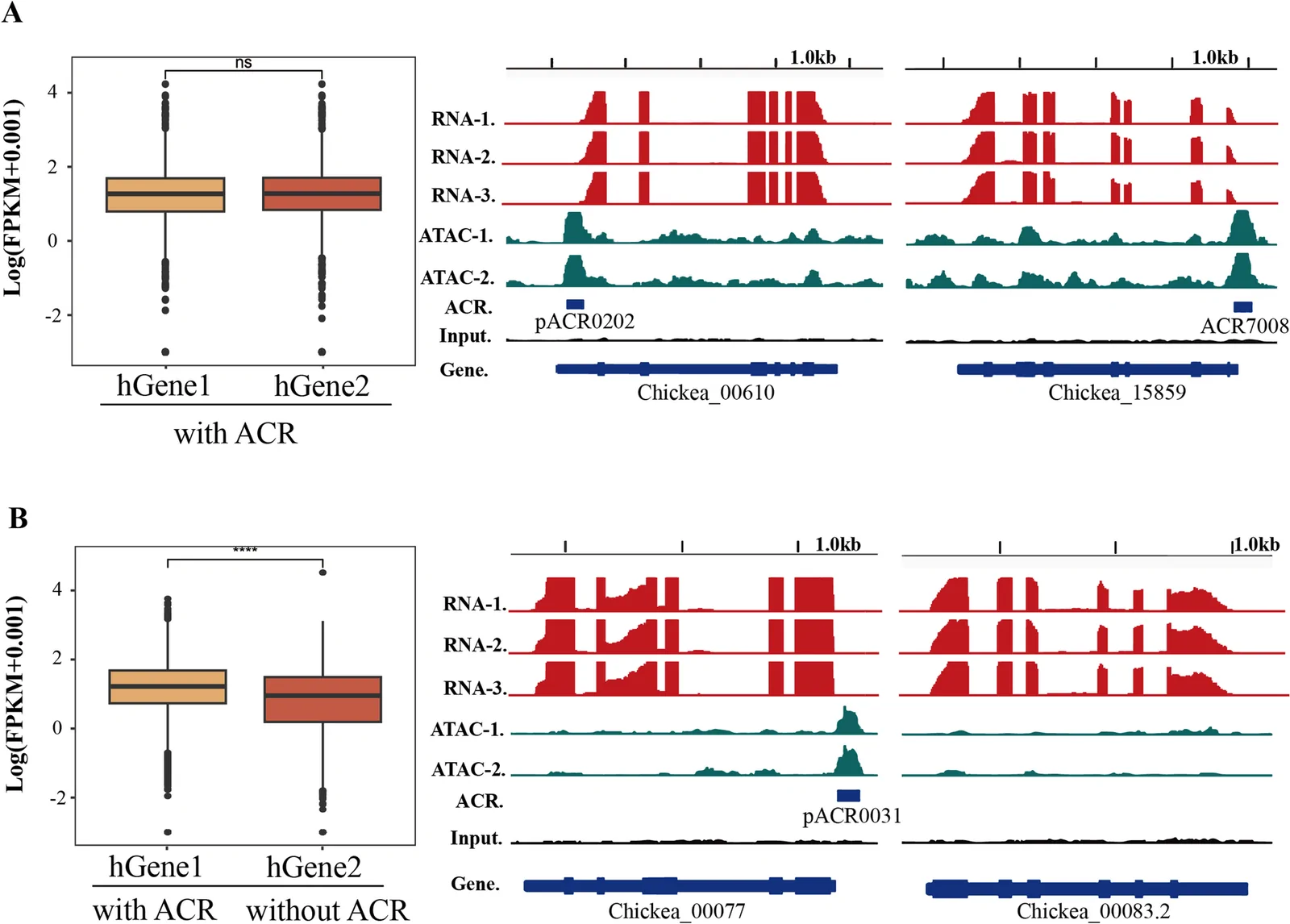
Potential influences of pACRs on homologous gene (hGene) expression. **A** Expression levels of hGenes associated with ACRs show no significant difference (left panel) (Wilcoxon test, n.s.). Examples of hGenes are both associated with ACRs in the IGV screenshot (right panel). **B** Expression levels of hGenes in which one does not associate with an ACR (left panel). Examples of the hGene pairs with or without ACRs (right panel) (Wilcoxon test; *p* < 0.0001)

Gene duplications are prevalent during whole genome duplication, resulting in the divergence of the homologous genes (hGenes). Indeed, during the duplication of hGenes, the gain and loss of their corresponding *cis*-regulatory sequences, such as the promoter, could also affect their neofunctionalization [38, 39]. The hGene pairs were derived from the ACR-associated genes (E-value < 1e-5) (Table S7). Both hGenes associated with ACRs showed no significant difference in their gene expression, while the expression of the hGene without ACRs was significantly lower than that of its hGene with ACRs (Fig. 5). Furthermore, the absence of ACRs in hGenes also affected their expression levels, which aligns with the global positive correlation observed between chromatin accessibility and gene expression (Table S7, Figure S7). This observation suggests an important role for ACRs in maintaining hGene expression during the progress of hGene duplication.

## Discussion

In this study, ATAC-seq has been employed for genome-wide characterization of ACRs in chickpea. The results revealed that ACRs were positively correlated with their associated gene expression, and were also enriched with many conserved TF motifs, suggesting that ACRs are important for gene regulation by acting as *cis*-regulatory sequences. In addition, ACRs were shown to be associated with active histone marks (e.g. H3K4me3 and H3K27ac) in the regulation of gene transcription. Finally, the presence or absence of ACRs could influence the expression levels of hGenes. Taken together, these data could be considered valuable genomic resources for future studies on gene regulation in chickpea.

ATAC-seq has been widely used as an efficient tool for gene regulation studies, such as TF-DNA interaction analysis and promoter-enhancer prediction in plants. For example, HSFA1a played an important role in the dynamic formation of promoter-enhancer contacts near the ACRs in response to heat stress in tomato, by ATAC-seq and other methods [40]. A total of 446 regeneration-related core TFs are associated with the open chromatin regions in wheat [7]. Moreover, ATAC-seq is currently being used to study a variety of plants, such as *Arabidopsis thaliana* [11, 41], maize [42–44], cultivated soybean [17, 43, 45–48], rice [49], grape [50], tomato [51] and the medicinal plant, *Andrographis paniculata* [52].

The function of distal ACRs has received much attention in recent investigations, as they may serve as enhancer-like elements that could mediate contact with proximal *cis*-regulatory sequences to promote gene expression [53, 54]. Similar to the proximal ACRs, the distal regions also contained conserved TF-binding motifs (e.g. AP2/ERF, bHLH, bZIP, etc.), through which the expression levels of genes could be precisely controlled, thereby affecting various biological processes in plants [55]. Therefore, several reports have indicated that variants in these non-coding regions, such as patch chromatin variants, single nucleotide polymorphisms, indels, etc., affect the domestication of the plants and their final phenotype [39, 45]. This phenomenon has been reported in plants such as wheat [21], tomato [38], maize [42], etc. In this study, several cases of gain and loss of ACRs associated with hGenes were observed, which significantly affected gene expression. It was suggested that the non-coding ACRs may also play an important role in the adaptation and domestication of chickpea, but further investigation is required to confirm this hypothesis. Regarding the chromatin signatures of these elements, it is noteworthy that many identified dACRs in chickpea lack typical enhancer-associated histone marks. Unlike animal enhancers, which exhibit abundant histone modification signals, most plant dACRs are largely unmodified, a phenomenon supported by previous studies [43]. This suggests that chromatin accessibility itself may be a more primary indicator of regulatory potential in chickpeas than traditional histone signatures.

## Conclusion

In this study, we have provided a comprehensive overview of ACRs and their genomic features across the chickpea genome, using ATAC-seq and histone ChIP-seq methods. Furthermore, this study has shown that ACRs play important roles in gene regulation and potential roles in plant domestication, and our data provide an important resource for further exploration of the chickpea genome.

## Supplementary Information

Supplementary Material 1.

Supplementary Material 2.

## Data Availability

The raw data in this study was submitted to The China National Center for Bioinformation (https://ngdc.cncb.ac.cn/gsa/) under the BioProject accession PRJCA058038.
