# Supplementary material for: Genome-wide characterization of the accessible chromatin regions in chickpea
Source: BMC Genomics. 2026 Jul 3;27:780. doi: 10.1186/s12864-026-13136-5 (PMC13602502; doi:10.1186/s12864-026-13136-5)
Supplement: Supplementary file 1 [file 12864_2026_13136_MOESM1_ESM.docx]

**Sup-Figure**

**Figure S1. Pearson correlation between two replicates of ATAC-seq data.**

**Figure S2. ATAC-seq quality assessment.**

(A-B) Insert-size distribution for replicates ATAC1 and ATAC2. Both plots show the characteristic nucleosome-free region (NFR) peak (<100 bp) and a clear mono-nucleosome peak (~180-240 bp).(C) TSS enrichment profiles for ATAC1 and ATAC2. Both replicates show a sharp signal peak at the transcription start site (TSS), flanked by nucleosome-depleted valleys. The signal is plotted in a ±2.0 kb window centered on the TSS.(D) Fingerprint plot for ATAC1 and ATAC2. The strong deviation of both replicates from the diagonal line indicates high signal enrichment in open chromatin regions (peaks) relative to the genomic background.

**Figure S3.ATAC-seq replicate quality metrics and concordance.**

(A-B) Cross-correlation plots for replicates ATAC1 and ATAC2. Both replicates show a strong enrichment peak at the fragment length (~110-120bp) and high signal-to-noise ratios, reflected in high Normalized Strand Coefficient (NSC ≈ 1.16) and Relative Strand Correlation (RSC ≈ 0.99) scores.(C) Fraction of Reads in Peaks (FRiP) scores for ATAC1 (0.653) and ATAC2 (0.654), indicating very high and consistent signal enrichment.(D) FRiP concordance scatter plot between replicates. The point's position on the diagonal line confirms the high reproducibility of signal enrichment between the two biological replicates.

**Figure S4.Aggregated TF footprint profiles for enriched motifs.**

Aggregated Tn5 insertion profiles centered on the binding sites of three representative TF motifs. (A) Motif 0402. (B) TFmatrixID_1238. (C) Motif 1427. All three profiles display a characteristic signal depression (footprint) at the motif center (0 bp), consistent with TF occupancy and protection from Tn5 cleavage.

**Figure S5. Pearson correlation among the histone modification ChIP-seq data.**

**Figure S6. Histone modification features of ACRs in chickpea genome genome.**

(A) Number of H3K4me3 and H3K27ac in each chromosome. (B) Genomic distribution of H3K4me3 and H3K27ac ChIP-seq enriched peaks, including Promoter, Downstream, Intergenic, and Exon.

**Figure S7.Genome-wide association between chromatin accessibility and gene expression.**

(A, B) Scatter plots showing the Spearman correlation between DESeq2-like normalized gene expression (expr_log1p_NormCounts) and log1p-transformed ATAC-seq signal at (A) TES (Transcription End Site, ±2 kb) and (B) TSS (Transcription Start Site, ±2 kb) for 17,161 genes. Correlation coefficients (r) and p-values are indicated.(C) Box plot comparing ATAC-seq accessibility (Log2(ATAC-seq Score + 1)) across genes grouped into High, Mid, and Low expression levels. To maintain consistency with analyses in the main manuscript (e.g., Fig. 1B, Fig. 3A), these groups are defined by FPKM. The overall significance was determined by a Kruskal-Wallis test (p < 2.2e-16), and asterisks (****) indicate significant pairwise comparisons (p < 0.0001, Wilcoxon test).
